# A Novel Sutureless Integrated Stented (SIS) Graft Prosthesis for Type A Aortic Dissection: A Pilot Study for a Prospective, Multicenter Clinical Trial

**DOI:** 10.3389/fcvm.2021.806104

**Published:** 2022-02-08

**Authors:** Lu Dai, Jiawei Qiu, Rui Zhao, Fangfang Cao, Juntao Qiu, De Wang, Shuya Fan, Enzehua Xie, Jian Song, Cuntao Yu

**Affiliations:** ^1^Department of Aortic Surgery, National Center for Cardiovascular Disease, Peking Union Medical College, Fuwai Hospital, Chinese Academy of Medical Sciences, Beijing, China; ^2^Adult Surgical Intensive Care Unit, National Center for Cardiovascular Disease, Peking Union Medical College, Fuwai Hospital, Chinese Academy of Medical Sciences, Beijing, China

**Keywords:** type A aortic dissection, sutureless integrated stented graft prosthesis, frozen elephant trunk, mortality, circulatory arrest time, deep hypothermia, surgery simplification

## Abstract

**Aims:**

Various kinds of surgical strategies and prostheses have been advocated to improve short-term and long-term outcomes in type A aortic dissection (TAAD). Large-scale repair of the pathological aorta is hard to generalize due to complex procedures. We aimed to investigate the performance, effectiveness and safety of a novel Sutureless Integrated Stented (SIS) graft prosthesis in TAAD patients undergoing total arch replacement (TAR) and frozen elephant trunk (FET) implantation surgery.

**Methods:**

All patients admitted to Fuwai Hospital were prospectively screened. Urgent or scheduled surgery was arranged for eligible patients. The primary endpoint was operative mortality. Key secondary endpoints included stroke, spinal cord injury, unexpected aortic reoperation, and 1-year survival. Discharged patients were followed up with computed tomography angiography and transthoracic echocardiography at 3 months, 6 months, and 1 year after surgery. Performance, effectiveness and safety analyses were performed in those patients.

**Results:**

Between August 1 and September 3, 2020, ten TAAD patients were enrolled in this study and successfully implanted with the SIS graft prosthesis. The median (IQR) age was 56.50 (43.75, 66.75) years (range from 31 to 75), and seven patients were male (70.0%). All patients underwent ascending aorta replacement + TAR + FET and additional procedures when necessary. The median (IQR) operation time, cardiopulmonary bypass time and cross clamp time were 270.50 (218.50, 312.50), 110.00 (88.00, 125.75), 69.50 (51.25, 82.75) min, respectively. Of note, the median (IQR) circulatory arrest time was 9.00 (8.00, 9.00) min (range from 4 to 12). The median (IQR) lowest nasopharyngeal temperature was 26.75 (25.98, 27.67) °C. Follow-up was 100% completed. During the 1-year follow-up, no patients died, no severe adverse events occurred, and rate of freedom from aortic reintervention was 100%.

**Conclusions:**

The SIS graft prosthesis was implanted in a novel sutureless way, which simplified the surgical procedure, shortened the circulatory arrest time and avoided deep hypothermia. The preliminary clinical outcomes and follow-up outcomes demonstrated the effectiveness and safety of this prosthesis. A large-scale trial is being conducted to further assess these findings.

## Introduction

Aortic dissection (AD) is a fatal disease with high mortality worldwide. It is well known that the patient population dying from AD is composed mainly of individuals with type A aortic dissection (TAAD). Hence, reducing the mortality of TAAD patients is a key approach to improve the prognosis of AD patients. To date, surgery is still the mainstay of treatment for TAAD patients. However, surgical strategies vary according to the extent of the dissecting aorta and the surgeon's treatment concept. For the aortic arch, the best practice guideline is still controversial. Total arch replacement (TAR) combined with frozen elephant trunk (FET) implantation has been applied in TAAD cases for almost 20 years in China (1). In recent years, more experts abroad have advocated the application of this strategy for TAAD patients with aortic arch involvement (2). However, this procedure is complex and difficult to carry out in low-volume centers, which limits the expansion of urgent TAAD treatment coverage.

Currently, a variety of prostheses developed by different centers are used for TAR+FET (3, 4). However, none of these surgeries can be completed without the hypothermia circulatory arrest technique, which has been applied for over 40 years as a protective measure for vital organs (5). In earlier years, it was regarded as the safest technique to assist surgeons in performing aortic arch surgeries. Over time, researchers gradually found that the time of circulatory arrest (CA) and temperature of hypothermia circulatory arrest were correlated with postoperative complications and prognosis.

Hence, simplifying the TAR+FET procedure and reducing complications caused by CA and hypothermia circulatory arrest have become a new complex issue. To address this challenge, a new prosthesis called the Sutureless Integrated Stented (SIS) graft prosthesis was developed (6, 7); it is characterized by the integration of a 4-branched vascular graft, sutureless device and FET. With this prosthesis, distal anastomosis can be completed in a sutureless manner. This study, which was a pilot study for a prospective, multicenter clinical trial, aimed to preliminarily investigate the performance, effectiveness and safety of the SIS graft prostheses in TAAD patients undergoing TAR+FET surgery.

## Materials and Methods

### Study Design and Participants

This study was approved by the Ethics Committee of Fuwai Hospital, Chinese Academy of Medical Sciences (approval number 2019-1241) and was preregistered on chictr.org.cn (unique identifier ChiCTR2000032264) on April 4, 2020 (URL: http://www.chictr.org.cn/showproj.aspx?proj=51524). This study followed the precepts of Good Clinical Practice and the Declaration of Helsinki.

Since the use of a controlled study design involving different FET prostheses was judged to be ethically infeasible by our ethics committee due to surgical risk differences, a single-arm design was finally selected. Patients admitted to Fuwai Hospital, Chinese Academy of Medical Sciences and Peking Union Medical College were screened for eligibility according to the inclusion and exclusion criteria (see the Supplementary Material, Supplementary Table S1). Before surgery, the patients were fully informed by the investigators about the study and then decided themselves whether to participate. Written informed consent was signed and kept in duplicate. Enrolled patients were assigned to urgent or scheduled surgery and use of the SIS graft prosthesis. Interventions were provided by surgeons in our center. Discharged participants were contacted by telephone 1 month after surgery and were asked to return to the hospital 3, 6, and 12 months after surgery. Inquiry, physical examination, aortic computed tomography angiography (CTA) examinations and transthoracic echocardiography were performed during the return visit.

### Sutureless Integrated Stented Graft Prosthesis

**Figure 1:**
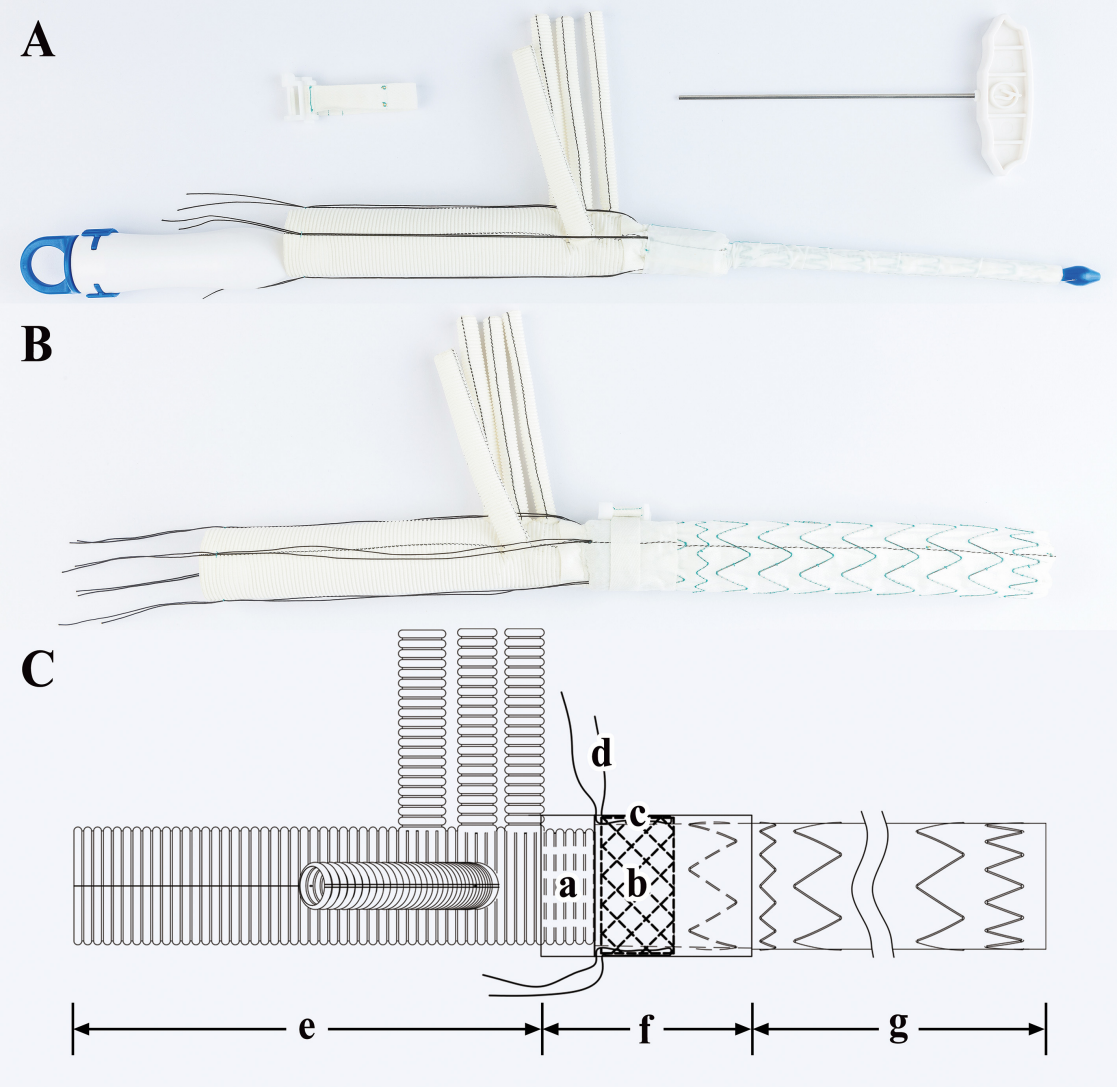
Sutureless Integrated Stented (SIS) graft prosthesis**. (A)** Real product image of the SIS graft prosthesis before release: a, Delivery system; b, Vascular graft; c, Sutureless belt; d, Wrench. **(B)** Real product image of the SIS graft prosthesis after release. **(C)** Design drawing of the SIS graft prosthesis: a, Terylene vascular graft with screw threads; b, Sutureless elastic support annulus; c, Terylene without screw threads; d, Restraint sutures; e, Terylene vascular graft region; f, Sutureless region; g, Stented-graft region.

The SIS graft prosthesis (Beijing Percutek Therapeutics Inc., Beijing, China) consists of a vascular graft and sutureless belt. The vascular graft integrates a 4-branched vascular graft, a sutureless elastic support annulus and a FET (Figure 1). From the proximal end to the distal end, it can be divided into three regions: the terylene vascular graft region, sutureless region and stented-graft region. The sutureless region is multilayered, and from the inner layer to the outer layer, it consists of a layer of terylene with screw threads, a sutureless elastic support annulus and a layer of terylene without screw threads. The sutureless elastic support annulus and stented graft are composed of nitinol and are able to self-expand after release. Before deployment, they are compacted and attached to the delivery system. Four silk sutures are evenly preset along the circumferential direction of the sutureless elastic support annulus. After pulling and tying these restraint sutures, the annulus is compressed in the axial direction to obtain a stronger supporting force. The sutureless region and stented-graft region are compressed in advance. Subsequently, they are implanted into the descending aorta by the delivery system and then released when managing the aortic arch and descending aorta. Another four silk sutures can be applied to the proximal end of the descending aorta and tied with restraint sutures to avoid migration of the vascular graft. On the area corresponding to the sutureless region, the sutureless belt can be wrapped around the adventitia of the aorta and then fastened by turning a ratchet with a wrench. Next, the graft is tightly attached to the autologous aortic wall such that anastomosis of the 4-branched vascular graft, FET and descending aorta is no longer needed. The final sutureless region from the inner layer to the outer layer comprises a layer of terylene with screw threads, a sutureless elastic support annulus, a layer of terylene without screw threads, autologous aortic wall and a sutureless belt. To prevent excessive fastening and necrosis of the aortic wall, we established a maximum turning force. When the turning force reaches the maximum value, the ratchet just slips. Technically, the SIS graft prosthesis can shorten the CA time and simplify surgical procedures due to their integrated and sutureless design.

### Intervention Procedure

**Figure 2:**
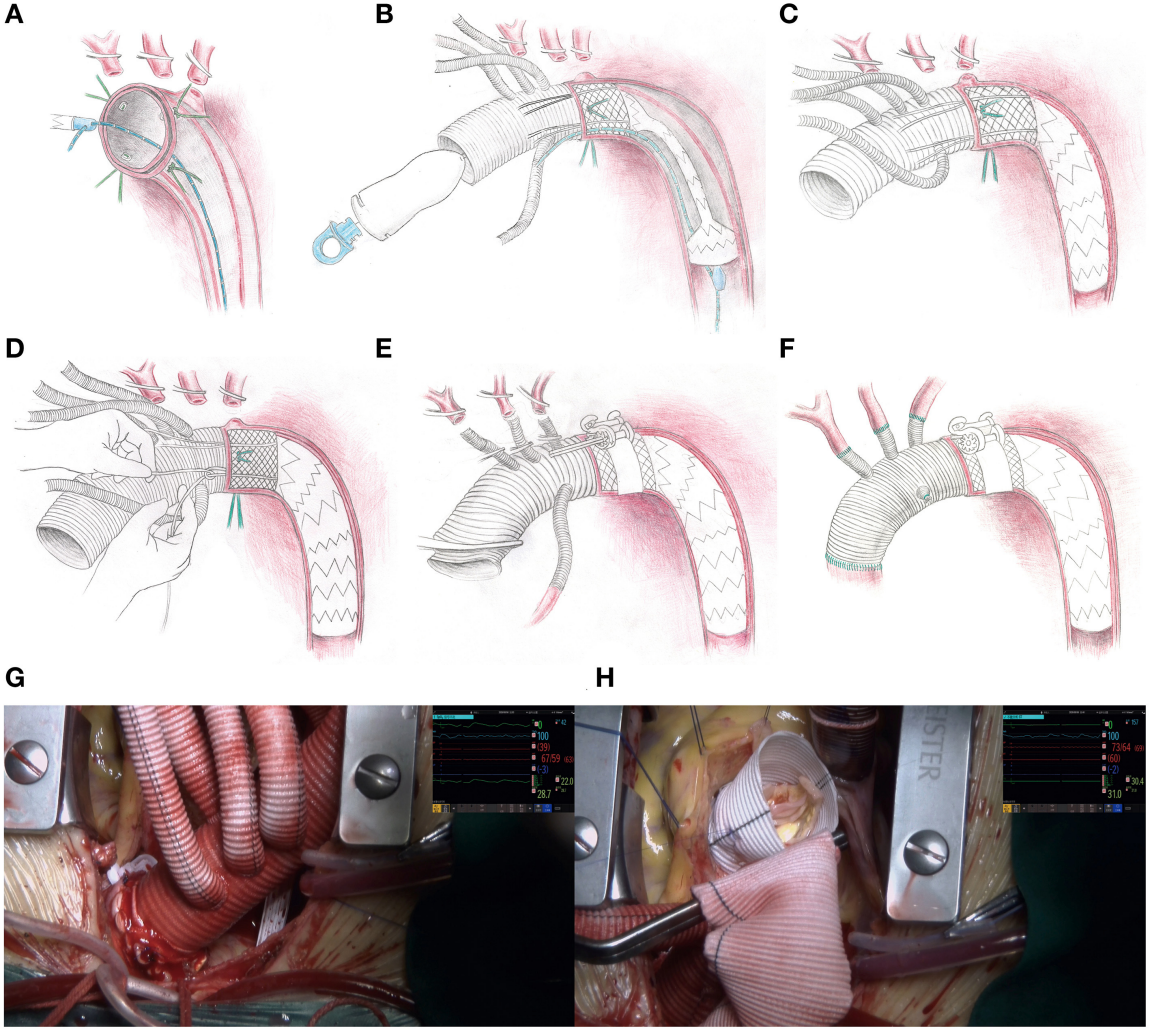
Operative procedure for the SIS graft prosthesis and video screenshots of the operation. **(A)** The aortic arch is dissected transversely, four sutures are applied evenly around the aortic wall, and a retrogradely inserted guidewire is threaded through an eyelet on the distal end of the delivery system. **(B)** The sutureless region and stented-graft region are deployed into the descending aorta over the guidewire and released by drawing out the pull ring on the proximal end of the delivery system. **(C)** The guidewire and delivery system are removed from the femoral access and the proximal orifice of the 4-branched vascular graft trunk, respectively. **(D)** The restraint sutures are securely tightened and tied *in situ*, and then tied with the corresponding silk sutures already threaded on the aortic wall. **(E)** The perfusion to the lower part of the body is restarted via the femoral cannula. On the area corresponding to the sutureless elastic support annulus, a sutureless belt is looped around the aortic adventitia and then fastened by truning the ratchet with the wrench. **(F)** The supra-aortic vessels and ascending aorta are anastomosed. **(G)** The appearance of the distal anastomosis site after fastening the sutureless belt. **(H)** The operative field is clear without bleeding from the distal anastomosis site when performing the David procedure.

Considering the heterogeneity of patients, the operative strategy must be individualized. The procedure is roughly described below (Figure 2).

Each patient was anesthetized in the supine position. A guidewire was inserted retrogradely into the aortic arch through the true lumen under transesophageal echocardiographic guidance via femoral access. Cardiopulmonary bypass was instituted by cannulation of the femoral artery with one end of a Y-shaped aortic cannula and right atrium with a venous single two-stage cannula. During the systemic cooling period, concomitant aortic root procedures (e.g., Bentall, adventitial inversion technique) or other procedures (e.g., coronary artery bypass grafting) were performed if necessary. An arch-first strategy is preferred in our center. A target nasopharyngeal temperature of 28°C was established, and the arch procedure began when the temperature reached that target, the arch procedure began. The left common carotid artery was first clamped and resected, and a catheter was introduced into the distal left common carotid artery with a flow rate of 3 mL/kg/min. Second, the femoral artery cannula was cross clamped, the CA was initiated, the innominate artery was clamped and resected, the other end of the Y-shaped aortic cannula was inserted into the distal innominate artery with a flow rate of 12 mL/kg/min and bilateral anterograde selective cerebral perfusion was established. Cerebral oxygen saturation was monitored by near infrared spectroscopy. Third, the left subclavian artery was clamped to avoid a steal phenomenon and to maintain a clear operative field. Next, the aortic arch was incised longitudinally up to the origin of the left subclavian artery (Ishimaru Zone 2) [in a modified procedural plan, the stopping site was moved to the origin of the left common carotid artery (Ishimaru Zone 1)] (8, 9). At the end of this incision, the aortic arch was dissected transversely. Four 10# silk sutures were evenly applied around the aortic wall, two of which were at the highest and lowest positions. The previously positioned guidewire was threaded through an eyelet at the distal end of the delivery system. The sutureless region and stented-graft region were smoothly deployed into the descending aorta over the guidewire after proper bending of the starting segment of the stent to fit the distal arch anatomy. When the sutureless elastic support annulus and stent were located properly, they were released by drawing out the pull ring on the proximal end of the delivery system. Then, the guidewire and delivery system were removed from the femoral access and the proximal orifice of the 4-branched vascular graft trunk, respectively. The four restraint sutures were securely tightened and tied *in situ*, and then tied with the corresponding silk sutures already threaded on the aortic wall. The proximal end of the terylene vascular graft and its four branches were cross clamped, and the femoral artery cannula clamp was released. Perfusion to the lower part of the body was then restarted *via* the femoral cannula, which means CA was over. In the area corresponding to the sutureless elastic support annulus, the sutureless belt was looped around the aortic wall and then fastened by turning the ratchet with the wrench. The left common carotid artery, left subclavian artery and innominate artery were anastomosed to branches on the greater curvature of the terylene vascular graft by continuous stitching of a 5-0 polypropylene suture. The proximal end of the terylene vascular graft was cut into suitable lengths and shapes and then anastomosed to the native aortic root or aortic root graft using continuous stitching of a 5-0 polypropylene suture. The previous end of the Y-shaped aortic cannula was transferred from the innominate artery to the 4th branch arising from the anterior wall of the terylene vascular graft to achieve antegrade perfusion assistance. The operation was completed after a routine hemostasis procedure. A detailed animation of the SIS graft prosthesis and a video of implantation of the SIS graft prosthesis in two patients can be viewed in the Supplementary Material or Figshare (10).

### Endpoints and Definitions

The primary endpoint was operative mortality, which was defined as all-cause deaths occurring before final hospital discharge (in-hospital mortality) or within 30 days of surgery (30-day mortality), including transfers to other acute care facilities according to the definition of the Society of Thoracic Surgeons. Late mortality was defined as all-cause death during the follow-up period. Stroke was defined as a postoperative new-onset focal lesion of the brain detected by computed tomography (CT) or magnetic resonance imaging (MRI) or documentation by a neurologist. Spinal cord injury was evaluated according to the ASIA (American Spinal Injury Association) Impairment Scale (AIS) (11). Permanent spinal cord injury was defined as irreversible paraplegia (AIS Grade A, B, or C at the time of final discharge, including transfers to other acute care facilities) (12). Pneumonia was defined as positive pathogenic detection in sputum samples or blood samples. Gastrointestinal bleeding was defined as positive results after a gastric occult blood test or fecal occult blood test. Aortic reintervention was defined as open surgical aortic repair or endovascular aortic repair postoperatively.

The definition of successful medical device implantation is summarized in the Supplementary Material (Supplementary Table S2). The definition of ischemic hepatitis is shown in the Supplementary Material (Supplementary Table S3) according to the revised Henrion's criteria (13, 14). Acute kidney injury (AKI) is defined and staged in the Supplementary Material (Supplementary Tables S4, S5) according to the Kidney Disease: Improving Global Outcomes (KDIGO) Clinical Practice Guidelines for Acute Kidney Injury (abbreviated as KDIGO criteria) (15). The stages of AD are classified in the Supplementary Material (Supplementary Table S6) according to the new International Registry of Acute Aortic Dissection (IRAD) classification system (16). Additional information on dissection anatomy and end-organ malperfusion is presented according to the TEM classification (17). The German Registry of Acute Aortic Dissection Type A (GERAADA) score is applied to predict operative mortality (18, 19).

The Fu Wai classification scheme developed by our center has been described previously (20, 21). In brief, Type A involves the ascending aorta and is restricted to the aorta proximal to the innominate artery. Type B is limited to the thoracic descending aorta, which may extend into the abdominal aorta or even iliac arteries. Type C involves the aortic arch, regardless of whether the ascending aorta/descending thoracic aorta is involved. Type C includes three subtypes. Type Ct (C total) involves the total aortic arch. Type Cp (C proximal) involves the proximal aortic arch, including only the innominate artery or innominate artery + left common carotid artery. Type Cd (C distal) involves the distal aortic arch, including only the left subclavian artery or left subclavian artery + left common carotid artery. Type D is limited to the abdominal aorta, which may extend into the iliac arteries.

### Data Collection

According to the protocol, all data related to this study were collected prospectively and saved in an electronic medical record system for assurance of traceability. Then, all data were extracted from the electronic medical record system and classified into preoperative, intraoperative and postoperative data. Follow-up data were extracted from telephone records, outpatient records and imaging reports. All variables were reported according to the Standards of Reporting in Open and Endovascular Aortic Surgery (STORAGE) guidelines (22).

### Statistical Analysis

Descriptive statistical analyses were performed for presentation of the variables for the present study. Categorical variables are presented as frequencies and percentages. Continuous variables are presented as the medians and interquartile ranges (25% [quartile 1] to 75% [quartile 3]). All data were imported into R software for Windows version 4.0.5 (The R Foundation for Statistical Computing, Vienna, Austria) for statistical analysis.

## Results

**Figure 3:**
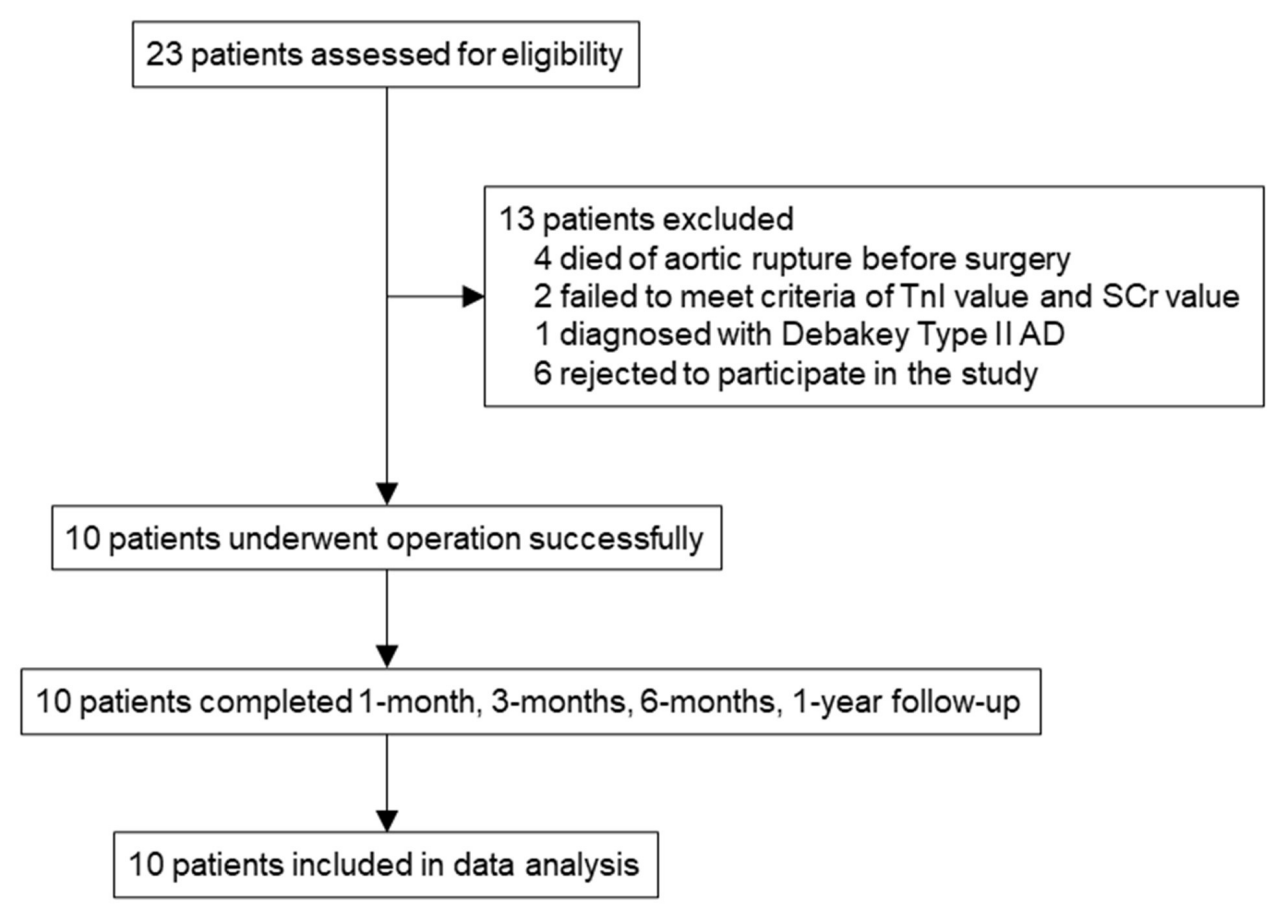
Trial profile. SCr, serum creatine; TnI, troponin I; AD, aortic dissection; CTA, computed tomography angiography; TTE, transthoracic echocardiogram.

Between August 1, 2020, and September 3, 2020, 23 TAAD patients admitted to Fuwai Hospital, Chinese Academy of Medical Sciences and Peking Union Medical College were screened (Figure 3). Finally, 10 patients were enrolled in this study. Patient characteristics and preoperative examinations are presented in Tables 1, 2, and Supplementary Table S7. The median (IQR) age was 56.50 (43.75, 66.75) years (range from 31 to 75), and 7 patients were male (70.0%). AD was acute or hyperacute in 7 (70.0%) patients. Three (30.0%) patients were aortic-root-aneurysm-based AD or ascending-aorta-aneurysm-based AD. Using the Fu Wai classification scheme, nine (90.0%) patients were diagnosed with Type Ct AD, and the remaining one (10.0%) patient was diagnosed with Type A combined with Type Cd AD.

**Table 1:** Baseline characteristics.

**Variables**	**Value (*n* = 10)**
Age (year)	56.50 (43.75, 66.75)
Male	7 (70.0)
Height (cm)	170.00 (162.25, 175.75)
Weight (kg)	69.50 (60.00, 80.00)
Admission during acute or hyperacute stage	7 (70.0)
History	
High blood pressure	7 (70.0)
Diabetes	0 (0.0)
Coronary artery disease	4 (40.0)
Valvular heart disease	0 (0.0)
Hyperlipidemia	4 (40.0)
Chronic obstructive pulmonary disease	0 (0.0)
Cerebral hemorrhage	0 (0.0)
Cerebral infarction	0 (0.0)
Severe liver disease	0 (0.0)
Chronic kidney disease	0 (0.0)
Peripheral vascular disease	0 (0.0)
Severe carotid artery stenosis	0 (0.0)
Malignancy	0 (0.0)
Congenital heart disease	1 (10.0)
Aortic coarctation	0 (0.0)
Marfan syndrome	2 (20.0)
Loeys-Diets syndrome	0 (0.0)
Ehlers-Danlos syndrome	0 (0.0)
Familial genetic aortic dissection	0 (0.0)
Takayasu arteritis	0 (0.0)
Iatrogenic aortic dissection	0 (0.0)
Atherosclerosis	4 (40.0)
Comorbid aortic aneurysm	3 (30.0)
Bicuspid aortic valve	1 (10.0)
Previous cardiac surgery	1 (10.0)
Smoking	6 (60.0)
Drinking	2 (20.0)
GERAADA score (%)	17.90 (16.33, 24.80)

*Data are presented as n (%) or median (25% [quartile 1] to 75% [quartile 3]). GERAADA, German Registry for Acute Type A Aortic Dissection*.

**Table 2:** Preoperative examinations.

**Variables**	**Value (*n* = 10)**
Laboratory test	
WBC (×10∧9/L)	10.15 (5.44, 10.93)
NEUT% (%)	72.45 (66.38, 81.50)
NEUT (×10∧9/L)	6.96 (3.72, 8.88)
RBC (×10∧12/L)	4.40 (3.74, 4.50)
Hgb (g/L)	136.50 (118.50, 139.75)
PLT (×10∧9/L)	202.50 (158.25, 244.50)
ALT (IU/L)	19.50 (13.75, 26.00)
AST (IU/L)	27.00 (21.00, 33.25)
TBIL (μmol/L)	12.68 (9.36, 17.94)
DBIL (μmol/L)	4.28 (2.98, 7.94)
BUN (mmol/L)	5.72 (4.20, 7.03)
SCr (μmol/L)	92.50 (72.87, 104.12)
CRP (mg/L)	11.37 (1.71, 15.27)
APTT (s)	37.85 (34.48, 38.67)
PT (s)	13.75 (13.27, 14.62)
FDP (μg/mL)	8.47 (5.64, 9.58)
D-dimer (μg/mL)	2.58 (1.93, 2.83)
TnI (ng/mL)	0.00 (0.00, 0.02)
MYO (ng/mL)	20.02 (15.73, 28.87)
CK-MB (ng/mL)	0.55 (0.32, 0.92)
PaO_2_ (mmHg)	96.40 (78.60, 114.50)
PaCO_2_ (mmHg)	39.85 (33.65, 42.65)
TTE	
Aortic insufficiency	
No	3 (30.0)
Mild	2 (20.0)
Between mild and moderate	1 (10.0)
Moderate	1 (10.0)
Between moderate and severe	2 (20.0)
Severe	1 (10.0)
Pericardial effusion	
No	8 (80.0)
Mild	1 (10.0)
Moderate	1 (10.0)
Severe	0 (0.0)
LVEF (%)	60.00 (60.00, 63.00)
Left atrial diameter (mm)	37.00 (30.00, 42.00)
Left ventricular end diastolic diameter (mm)	48.00 (47.00, 55.50)
Fu Wai classification	
Type A + Type Cd	1 (10.0)
Type Ct	9 (90.0)

*Data are presented as n (%) or median (25% [quartile 1] to 75% [quartile 3])*.

*WBC, white blood cell; NEUT, neutrophil; RBC, red blood cell; Hgb, hemoglobin; PLT, platelet; ALT, alanine aminotransferase; AST, aspartate aminotransferase; TBIL, total bilirubin; DBIL, direct bilirubin; BUN, blood urea nitrogen; SCr, serum creatine; CRP, C-reactive protein; APTT, activated partial thromboplastin time; PT, prothrombin time; FDP, fibrinogen degradation products; TnI, troponin I; MYO, myoglobin; CK-MB, creatine kinase-MB; PaO2, partial pressure of oxygen; PaCO2, partial pressure of carbon dioxide; TTE, transthoracic echocardiogram; LVEF, left ventricular ejection fraction*.

The SIS graft prosthesis was successfully implanted in all 10 patients. The operative details are outlined in Table 3. All patients underwent ascending aorta replacement + TAR + FET. The aortic root procedure varied depending on the severity of the aortic root. The median (IQR) operation time, cardiopulmonary bypass time and cross clamp time were 270.50 (218.50, 312.50), 110.00 (88.00, 125.75), and 69.50 (51.25, 82.75) minutes, respectively. Of note, the median (IQR) CA time was 9.00 (8.00, 9.00) minutes (range from 4 to 12). The median (IQR) lowest nasopharyngeal temperature was 26.75 (25.98, 27.67) °C. During the operation, 1 unit of platelets was transfused to every patient; only two patients received red blood cell transfusion (4 units each), and two patients received fresh frozen plasma (400 and 800 mL).

**Table 3:** Operative details.

**Variables**	**Value (*n* = 10)**
Surgery during acute or hyperacute stage	7 (70.0)
Cerebrospinal fluid drainage before surgery	0 (0.0)
Procedural duration	
Operation time (min)	270.50 (218.50, 312.50)
CPB time (min)	110.00 (88.00, 125.75)
Cross clamp time (min)	69.50 (51.25, 82.75)
Circulatory arrest time (min)	9.00 (8.00, 9.00)
Lowest nasopharyngeal temperature (°C)	26.75 (25.98, 27.67)
Artery cannulation site	
Axillary artery	3 (30.0)
Femoral artery	6 (60.0)
Ascending aorta	1 (10.0)
Cerebral perfusion	
Bilateral anterograde	10 (100.0)
Transfusion	
Platelets (Unit)	1.00 (1.00, 1.00)
Red blood cells (Unit)	0.00 (0.00, 0.00)
Fresh frozen plasma (mL)	0.00 (0.00, 0.00)
Concomitant operation	
Bentall	3 (30.0)
Mechanical valve	3 (30.0)
Valve-sparing root replacement	1 (10.0)
Reimplantation technique	1 (10.0)
Adventitial inversion technique	2 (20.0)
Ascending aorta replacement	10 (100.0)
Ascending aorta-femoral artery bypass	1 (10.0)

*Data are presented as n (%) or median (25% [quartile 1] to 75% [quartile 3]). CPB, cardiopulmonary bypass*.

**Figure 4:**
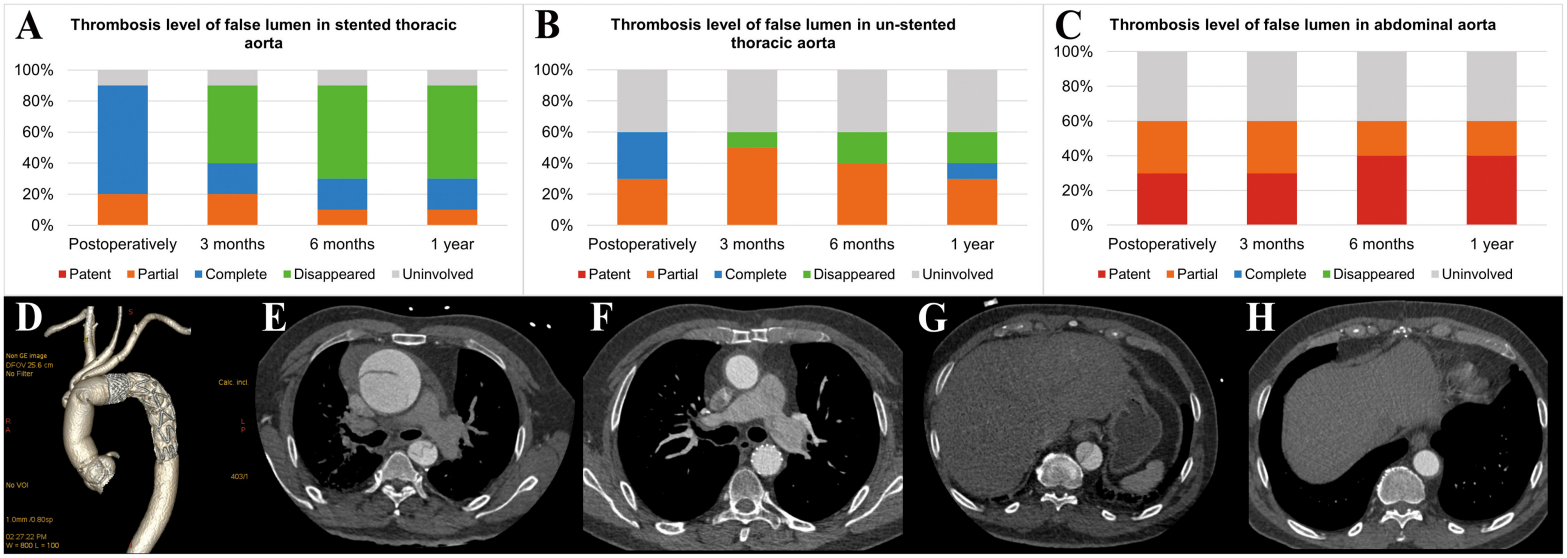
Aortic remodeling after surgery. **(A)** Proportions of different FL thrombosis levels in the stented thoracic aorta at different time points after surgery. **(B)** Proportions of different FL thrombosis levels in the unstented thoracic aorta at different time points after surgery. **(C)** Proportions of different FL thrombosis levels in the abdominal aorta at different time points after surgery. **(D)** The 3D reconstruction of aortic CTA shows the appearance of the SIS graft prosthesis after AAR+TAR+FET implantation surgery. **(E–H)** The axial view of aortic CTA presents comparisons of the aorta preoperatively and 3 months postoperatively in the same patient who underwent Bentall+AAR+TAR+FET implantation. **(E)** At the pulmonary artery bifurcation level, the intimal flap, TL and FL could be seen clearly in the ascending aorta and descending aorta preoperatively. **(F)** The ascending aorta was replaced with a terylene vascular graft, and the FL completely disappeared after FET implantation. **(G)** At the T10 level, the intimal flap, TL and FL could be seen clearly in the descending aorta preoperatively. **(H)** The FL disappeared in the unstented region by 3 months postoperatively. FL, false lumen; 3D, three-dimensional; CTA, computed tomography angiography; AAR, ascending aorta replacement; TAR, total arch replacement; FET, frozen elephant trunk; TL, true lumen.

Postoperative examinations results are shown in Table 4 and Figure 4. In all patients, prosthesis-related adverse events, the distal landing zone of FET and false lumen patency at different levels were evaluated by aortic CTA after removal of drainage tubes. No pseudoaneurysms in the anastomosis segment were found, and 2 (20.0%) patients developed type II endoleaks from intercostal arteries. The distal landing zone was located between the T6 and T10 levels, with most (60.0%) at T9. At the level of the stented thoracic aorta, the complete patency rate was 0.0%, and the partial patency rate was only 20.0%. At the level of the unstented thoracic aorta, 4 (40.0%) patients did not have AD preoperatively, and the remaining six (60.0%) patients showed complete or partial false lumen thrombosis. Distally, at the level of the abdominal aorta, of the six patients with AD, partial false lumen thrombosis occurred in three (30.0%) patients, and complete patency persisted in three (30.0%) patients.

**Table 4:** Postoperative examinations.

**Variables**	**Value**
Laboratory test before discharge (within 1 day)	
WBC (×10∧9/L)	8.62 (7.88, 10.01)
NEUT% (%)	75.80 (71.05, 79.32)
NEUT (×10∧9/L)	6.66 (5.64, 7.51)
RBC (×10∧12/L)	3.24 (3.03, 3.53)
Hgb (g/L)	98.00 (91.75, 107.75)
PLT (×10∧9/L)	305.00 (248.75, 372.25)
ALT (IU/L)	39.00 (16.75, 53.00)
AST (IU/L)	31.00 (20.75, 34.50)
TBIL (μmol/L)	9.10 (7.35, 10.79)
DBIL (μmol/L)	3.91 (3.06, 5.58)
BUN (mmol/L)	7.45 (5.06, 10.01)
SCr (μmol/L)	86.78 (80.54, 95.03)
CRP (mg/L)	11.39 (10.32, 13.01)
APTT (s)	43.00 (40.55, 47.88)
PT (s)	14.15 (13.62, 17.33)
FDP (μg/mL)	24.19 (17.54, 31.59)
D-dimer (μg/mL)	6.69 (5.15, 8.45)
TTE	
Lowest LVEF postoperatively (%)	54.50 (52.25, 55.00)
The last TTE before discharge	
LVEF (%)	58.50 (55.00, 61.75)
Left atrial diameter (mm)	37.50 (31.50, 42.00)
Left ventricular end diastolic diameter (mm)	47.00 (47.00, 52.75)
Aortic CTA	
Pseudoaneurysm in anastomosis segment	0 (0.0)
Endoleak of stent graft	
Type I	0 (0.0)
Type II	2 (20.0)
Type III	0 (0.0)
Type IV	0 (0.0)
Distal landing zone	
T6	1 (10.0)
T8	2 (20.0)
T9	6 (60.0)
T10	1 (10.0)

*Data are presented as n (%) or median (25% [quartile 1] to 75% [quartile 3])*.

*WBC, white blood cell; NEUT, neutrophil; RBC, red blood cell; Hgb, hemoglobin; PLT, platelet; ALT, alanine aminotransferase; AST, aspartate aminotransferase; TBIL, total bilirubin; DBIL, direct bilirubin; BUN, blood urea nitrogen; SCr, serum creatine; CRP, C-reactive protein; APTT, activated partial thromboplastin time; PT, prothrombin time; FDP, fibrinogen degradation products; TTE, transthoracic echocardiogram; LVEF, left ventricular ejection fraction; CTA, computed tomography angiography*.

Table 5 shows the in-hospital outcomes. No patients died after surgery, and no severe postoperative complications occurred. AKI was observed in 8 (80.0%) patients, but none required further continuous renal replacement therapy (CRRT), and their creatine levels returned to baseline before discharge. Of the four patients who underwent blood products transfusion postoperatively, one patient received 3 units of platelets and 6 units of red blood cells, one patient received 2 units of red blood cells, one patient received 4 units of red blood cells, and the remaining patient received 600 mL of fresh frozen plasma.

**Table 5:** In-hospital outcomes.

**Variables**	**Value**
Mechanical ventilation time (h)	12.50 (9.00, 17.00)
Postoperative drainage	
0–24 h (mL)	680.00 (547.50, 835.00)
24–48 h (mL)	365.00 (275.00, 572.50)
48–72 h (mL)	325.00 (255.00, 417.50)
Transfusion before discharge	
Platelets (Unit)	0.00 (0.00, 0.00)
Red blood cells (Unit)	0.00 (0.00, 1.50)
Fresh frozen plasma (mL)	0.00 (0.00, 0.00)
ICU stay (d)	4.50 (4.00, 6.00)
Complications	
Reoperation for bleeding	0 (0.0)
Reintubation	0 (0.0)
Tracheostomy	0 (0.0)
Stroke	0 (0.0)
Permanent spinal cord injury	0 (0.0)
Myocardial infarction	0 (0.0)
Pneumonia	1 (10.0)
Gastrointestinal bleeding	0 (0.0)
Ischemic hepatitis	1 (10.0)
New deep vein thrombosis of lower extremity	0 (0.0)
Pulmonary embolism	0 (0.0)
Pericardial tamponade	0 (0.0)
ECMO	0 (0.0)
IABP	0 (0.0)
Acute kidney injury	8 (80.0)
Stage 1	5 (50.0)
Stage 2	2 (20.0)
Stage 3	1 (10.0)
CRRT	0 (0.0)
Aortic reintervention	0 (0.0)
Death	0 (0.0)
Hospital stay (d)	17.00 (12.50, 20.50)

*Data are presented as n (%) or median (25% [quartile 1] to 75% [quartile 3])*.

*ICU, intensive care unit; ECMO, extracorporeal membrane oxygenation; IABP, intra-aortic balloon pump; CRRT, continuous renal replacement therapy*.

Follow-up outcomes are summarized in Table 6 and Figure 4. The survival rate at the 1-year follow-up was 100%. All patients were kept under close surveillance, and no patients ever experienced any major organ complications. Rate of freedom from prosthesis-related adverse events and aortic reintervention was 100%. No patients experienced pseudoaneurysms in the anastomosis segment. The FET position remained stable in all patients. Regarding the two patients with endoleaks, one case resolved spontaneously 6 months after surgery, and a gradual decrease in endoleak was noted in the remaining patient. At the stented thoracic aorta level, patients' numbers of complete reverse remodeling with disappearance of the false lumen increased and reached to 6 at the 6-month follow-up. Representative examples are shown in Figure 4. At the 1-year follow-up, only 1 patient remained in a state of partial false lumen thrombosis because of type II endoleak. In the unstented thoracic aorta, false lumen obliteration was achieved in 1 patient at the 3-month follow-up and in another patient at the 6-month follow-up. The rate of complete and partial thrombosis of the false lumen was 40% at the 1-year follow-up. In the abdominal aorta, the false lumen remained completely or partially perfused in 60% of patients as a consequence of the existence of secondary intimal tears until the 1-year follow-up. Regarding aortic diameter changes, there were no incidences of increasing false lumen diameters or total aortic diameters at any level during the 1-year follow-up.

**Table 6:** Follow-up outcomes.

**Variables**	**3-months value**	**6-months value**	**1-year value**
TTE			
LVEF (%)	60.50 (56.50, 62.00)	62.50 (60.25, 65.00)	61.00 (58.50, 65.00)
Aortic CTA			
Pseudoaneurysm in anastomosis segment	0 (0.0)	0 (0.0)	0 (0.0)
Endoleak of stent graft			
Type I	0 (0.0)	0 (0.0)	0 (0.0)
Type II	2 (20.0)	1 (10.0)	1 (10.0)
Type III	0 (0.0)	0 (0.0)	0 (0.0)
Type IV	0 (0.0)	0 (0.0)	0 (0.0)
Distal landing zone			
T6	1 (10.0)	1 (10.0)	1 (10.0)
T8	2 (20.0)	2 (20.0)	2 (20.0)
T9	6 (60.0)	6 (60.0)	6 (60.0)
T10	1 (10.0)	1 (10.0)	1 (10.0)
New aortic dissection	0 (0.0)	0 (0.0)	0 (0.0)
New intramural haematoma	0 (0.0)	0 (0.0)	0 (0.0)
New penetrating aortic ulcer	0 (0.0)	0 (0.0)	0 (0.0)
Aortic rupture	0 (0.0)	0 (0.0)	0 (0.0)
Total aortic diameter >60 mm in any segment	0 (0.0)	0 (0.0)	0 (0.0)
Total aortic diameter increase > 5 mm in any segment	0 (0.0)	0 (0.0)	0 (0.0)
FL diameter increase >5 mm in any segment	0 (0.0)	0 (0.0)	0 (0.0)
Medical device related adverse event	0 (0.0)	0 (0.0)	0 (0.0)
Aortic reintervention	0 (0.0)	0 (0.0)	0 (0.0)
Death	0 (0.0)	0 (0.0)	0 (0.0)

*Data are presented as n (%) or median (25% [quartile 1] to 75% [quartile 3])*.

*TTE, transthoracic echocardiogram; LVEF, Left ventricular ejection fraction; CTA, computed tomography angiography; FL, false lumen*.

## Discussion

To date, no consensus has been reached regarding the optimal strategy for maxmizing safety during aortic arch surgery, especially TAAD with arch involvement. As a more aggressive procedure, TAR+FET was thought to be associated with a higher mortality and poorer prognosis. According to previously published papers, in-hospital mortality associate with the FET procedure was reported to range from 2.1 to 21% (23, 24). These wide variations in mortality may be attributed to differences in experience in addition to the aggressiveness of the procedure. Dinato and associates divided their patients into two groups according to the operative date distribution (25). The results showed that earlier in-hospital mortality (from 2009 to 2014) was significantly higher than that later (from 2015 to 2018) (30.7 vs. 10%; *P* = 0.02) and indicated that operative outcomes improved as experience accumulated. In our review of FET-related research, most high-volume centers carried out approximately 50 FET procedures annually, and high-volume centers in China could perform more than 200 procedures annually; the outcomes were acceptable (26–31). However, low-volume centers performed significantly fewer procedures annually and reported poor prognoses. In the United States, a limited number of centers have tried to adopt the FET technique. In fact, the reason for the slow adoption and acceptance of this technique is not unpleasant outcomes but a lack of availability of commercial FET devices that can be widely used. Nevertheless, Roselli and associates demonstrated encouraging results using FETs and were optimistic about the application of the FET technique (32). In the J-ORCHESTRA study conducted in Japan, FET devices were used to treat more than 100 patients annually, and in-hospital mortality was lowered to 2.4% (33). Thus, low-volume centers may achieve improved surgical outcomes through experience accumulation.

Another controversy regarding TAR+FET is whether patients benefit from this extensive procedure. For TAAD patients, the ideal procedure is to obliterate the entire pathological aorta. However, primary surgery does not always achieve that goal, and reintervention is required if residual aortic remodeling is not satisfactory or new aortic events occur. In this setting, FET is designed with the aim of covering the entry of the proximal descending aorta and restoring the flow into the true lumen, resulting in thrombosis of the FL and better aortic remodeling. In Shrestha's previous work of 100 patients treated with FET (34), 24% of them underwent reoperations as a consequence of adverse aortic remodeling of the aortic arch and proximal descending aorta after limited repair of acute aortic dissection. These patients would not have needed this operation if they had received FET in the initial surgery. Inoue and colleagues reported that rates of freedom from dissection-related reoperation were significantly higher among patients with a thrombosed false lumen (100%) above the diaphragm level based on a median follow-up of 39 months (35). Such findings suggest that FET helps decrease the risk of reoperation. Moreover, data from Sino-RAD revealed a significantly younger onset age of TAAD in China compared with data from IRAD (36). The life expectancy of TAAD patients is at least 20 years in China and more than 10 years in Western countries. If treated less aggressively, many patients will require reoperation for the rest of their lives, and most of them will be in a worse clinical condition (i.e., advanced age or new comorbidities). Even worse, Shrestha and his colleagues proposed that a significant proportion of TAAD survivors would die from aortic-associated causes before reinterventions are performed (24). Specifically, in China, most patients are unwilling to accept a secondary surgery because they cannot afford the high treatment cost. Other reasons include fear of surgery and poor medical compliance behavior. This reminds us not only to care about surgical outcomes but also to consider socioeconomic factors and psychological experience and to provide health care in a more comprehensive manner to maximize the benefits to patients. Hence, staged operations are not applicable, at least in China, and FET is the best tool for achieving a preferred outcome (i.e., acceptable operative outcomes and prevention of reintervention).

Even though it is promising, FET still needs to be modified. Before commercial FET device was available, a “hybrid operation” was performed using a thoracic endovascular aortic repair (TEVAR) stent graft as the FET device, which resulted in problems such as unstable proximal fixation, distal migration, and type 1A endoleaks (37). Currently, common FET devices include the Chavan-Haverich graft (CURATIVE MEDICAL DEVICE GmbH, Dresden, Germany), Thoraflex Hybrid graft (Terumo, Glasgow, Scotland), E-vita OPEN graft (JOTEC, Hechingen, Germany), E-vita OPEN PLUS graft (JOTEC, Hechingen, Germany), E-vita OPEN NEO graft (JOTEC, Hechingen, Germany), J Graft Open Stent Graft (Japan Lifeline, Toda, Japan) and CRONUS graft (MicroPort, Shanghai, China). All grafts have a surgically sewn proximal end, which nearly eliminates the risk mentioned above. However, sutures are inevitable when fixing FET devices, which is time-consuming during CA. In addition, it is sometimes very difficult to expose the distal aorta and perform distal anastomosis (38); indeed, intraoperative bleeding at aortic anastomotic sites and increasing blood transfusion needs are foreseeable. Different wrapping techniques must be used to stop suture line bleeding from anastomotic sites. In recent years, some FET iterations with different designing purposes have been developed and applied (32, 39, 40). Surgical procedure simplification was our same opinion. However, considering distal aortic arch anastomosis, all these devices must be deployed with a running suture, which is not different from other FET devices described above. Time of CA is even longer because advancing the stent into the supra-aortic branches should be completed during CA in addition to distal arch anastomosis. To the best of our knowledge, we are the first to report an integrated graft that can achieve a “sutureless” method of distal aortic arch anastomosis. With the SIS graft prosthesis, a high anastomotic skill level is no longer needed when performing TAR+FET. Although similar operative strategies were used, our operation time, cardiopulmonary bypass time and cross clamp time were significantly shorter than those of many institutions (41, 42). Moreover, the blood transfusion need was low. In particular, the CA time was shortened by over 75% compared with that of other conventional FET procedures (41, 42). Deep hypothermia circulatory arrest was first proposed to avoid injury from long CA and provide multiple-organ protection. Now that a long CA time is not needed, we chose to apply a moderate hypothermia circulatory arrest strategy aimed at preventing complications caused by deep hypothermia circulatory arrest, and a shorter rewarming time definitely resulted in a shorter cardiopulmonary bypass time, which has been recognized as an independent risk factor for poor outcome (27, 31, 43).

Postoperative and follow-up outcomes were satisfactory. No patients died or required reintervention during the 1-year follow-up, and none encountered severe postoperative complications. FET-related complications, such as endoleaks, were also of concern. In his paper, Chu stressed the importance of paying attention to the adequacy of stent graft expansion due to the lower radial force characteristic of nitinol-ringed stents (44). We ensured full expansion soon after the release of the stent graft by transesophageal echocardiography, as also demonstrated by aortic CTA postoperatively. Although type I endoleaks were prevented, type II endoleaks from the intercostal artery were detected in 2 chronic TAAD patients. Auspiciously, the endoleak in one patient disappeared completely by the 6-month follow-up, and the endoleak in the other patient decreased gradually at every CT scan follow-up. Ius first reported his experience comparing three different FET prostheses, the Chavan-Haverich graft, E-vita OPEN graft, and Thoraflex Hybrid graft (45). In his study, the endoleak rate was 28% in all patients at the first CT scan follow-up and 22% in all patients at the last CT scan follow-up, both of which were higher than the rate in the present study. According to the subgroup analysis, the Thoraflex Hybrid graft had the lowest endoleak rates, namely 15 and 19% at the first and last follow-ups, respectively, which were similar to our results.

Many surgeons have discussed their concerns regarding the risk of spinal cord injury after implementation of the FET technique (46, 47), and most have advocated a higher distal landing zone in all patients and cerebrospinal fluid drainage in high-risk patients. We did not use cerebrospinal fluid drainage in any patient, and the distal landing zone distal to or equal to T8 accounted for 90% of all patients. No one experienced spinal cord injury, which may indicate that the distal landing zone may not be the sole or key element contributing to spinal cord injury development. This concept has been demonstrated by Hoffman and his colleagues by showing zero spinal cord injury occurrences after deployment of FET devices or extensive endovascular stent grafts between the T10 and T12 vertebral levels in all patients (48). Pacini and Leontyev both failed to identify a correlation between the distal landing zone level and the prevalence of spinal cord injury in their research (29, 49, 50). Mousavizadeh demonstrated statistically significant relationships between CA time and the development of postoperative stroke and spinal cord injury (51). Detailed results showed that as the CA time increased, the effect sizes for postoperative stroke and spinal cord injury were enhanced. As discussed above, our CA time was much shorter than those in many other studies, which may be helpful in preventing patients from experiencing spinal cord injury.

In the present study, 80% of patients were observed to develop acute kidney injury postoperatively, which was comparable to the rate in our prior reports (52). From our perspective, the reason for this high incidence of AKI was the high diagnostic sensitivity of the KDIGO criteria, especially for those with a normal baseline serum creatine. For example, serum creatine was 59.65 μmol/L in one of our subjects preoperatively, and the highest serum creatine was 168.51 μmol/L during hospitalization, which was categorized as stage 3 AKI but only exceeded the upper limit of normal for the serum creatine value by 25%. Moreover, various diagnostic criteria were applied in different studies, some of which did not even describe a diagnostic criterion but listed only the incidences of AKI in the results table. Thus, diversity of AKI incidence was inevitable.

Our study had some limitations. First, its sample size was small. However, the study had a prospective design so that we should comply with standardized protocols and inclusion and exclusion criteria. In addition, a subsequent large-sample, prospective and multicenter clinical trial is ongoing. Second, we did not have a control group with which to compare the study patients, as this was primarily a feasibility study. In fact, there have not been any FET-related randomized controlled trials to date. Each prosthesis was introduced into practice at different time points and was employed later than conservative procedures such as hemi-arch replacement and isolated ascending aorta replacement, rendering it difficult to balance surgical experience levels in a controlled design and causing it to be ethically judged as incomparable by our ethics committee due to surgical risk differences. Third, this was a single-center study, but we did not select mild cases subjectively. We tried to improve the reliability of our results and convinced other centers that multicenter trial is promising. Fourth, the surgeon in our center was on an early learning curve with this prosthesis, which indicated that these results could have been better. Fifth, even though all patients were stable at the 1-year follow-up, the follow-up period was relatively short, and midterm and long-term follow-ups are necessary.

## Conclusions

Preliminary clinical application of the SIS graft prosthesis met our expectation of shortening CA time, avoiding deep hypothermia circulatory arrest, and simplifying surgical procedure. Safety, effectiveness, feasibility, and performance of this prosthesis were initially verified. Longer-term follow-up and larger-sample trials are warranted to validate the safety and effectiveness of this device.

## Data Availability Statement

Data from this study are part of the ongoing prospective, multicenter clinical trial. Since the trial has not been completed, the data are now not publicly available. However, data are available from the corresponding author upon reasonable request, which must be submitted after obtaining permission from the Ministry of Science and Technology of the People's Republic of China according to the Regulations on Management of Human Genetic Resources promulgated since July 1, 2019. (URL: http://english.www.gov.cn/policies/latest_releases/2019/06/10/content_281476708945462.htm).

## Ethics Statement

The studies involving human participants were reviewed and approved by Ethics Committee of Fuwai Hospital, Chinese Academy of Medical Sciences. The patients/participants provided their written informed consent to participate in this study.

## Author Contributions

LD and CY designed the study. LD and JiQ enrolled the patients. CY performed the surgery. LD, JiQ, FC, and DW treated the patients. LD completed the follow-up work. LD, SF, EX, and JS collected and verified the data. LD and RZ analyzed and interpreted the data. LD, JiQ, and RZ drafted the paper. The corresponding author attests that all listed authors meet authorship criteria and that no others meeting the criteria have been omitted. All authors critically revised the manuscript for important intellectual content and agreed to submit the final version for publication, had full access to all the data in the study, took responsibility for the integrity of the data and the accuracy of the data analysis, and agreed to be accountable for all aspects of the work in ensuring that questions related to the accuracy or integrity of any part of the work were appropriately investigated and resolved.

## Funding

This work was supported by Beijing Science and Technology Project [Z191100007619042]; Capital Health Development and Scientific Research Foundation [2018-2-4035], CAMS Initiative for Innovative Medicine [2016-I2M-1-016] and Special Subject Development Foundation of Fuwai Hospital [2015-FWTS01]. The funders had no role in the design and conduct of the study; collection, management, analysis, and interpretation of the data; preparation, review, or approval of the manuscript; and decision to submit the manuscript for publication.

## Conflict of Interest

The authors declare that the research was conducted in the absence of any commercial or financial relationships that could be construed as a potential conflict of interest.

## Publisher's Note

All claims expressed in this article are solely those of the authors and do not necessarily represent those of their affiliated organizations, or those of the publisher, the editors and the reviewers. Any product that may be evaluated in this article, or claim that may be made by its manufacturer, is not guaranteed or endorsed by the publisher.
