# Supplementary material for: A Novel Sutureless Integrated Stented (SIS) Graft Prosthesis for Type A Aortic Dissection: A Pilot Study for a Prospective, Multicenter Clinical Trial
Source: Front Cardiovasc Med. 2022 Feb 8;8:806104. doi: 10.3389/fcvm.2021.806104 (PMC8860904; doi:10.3389/fcvm.2021.806104)

SUPPLEMENTARY MATERIAL

Contents

Supplemental Table S1: Eligibility Criteria…………………….…….2

Supplemental Table S2: Definition of successful medical device implantation…………………………………………………….….3

Supplemental Table S3: Definition of ischemic hepatitis………………4

Supplemental Table S4: Definition of AKI…………………………...5

Supplemental Table S5: Staging of AKI……………………………...6

Supplemental Table S6: Time phase of AD…………………………..7

Supplemental Table S7: Supplemental preoperative characteristics……8

Video 1: Detailed operation animation of the Sutureless Integrated Stented graft prosthesis…………………………………………...9

Video 2: Operation video of one acute type A aortic dissection patient and one chronic type A aortic dissection patient implanted with the Sutureless Integrated Stented graft prothesis…………………………..9

Supplemental Table S1: Eligibility Criteria

Inclusion Criteria	
1	Aged between 18 and 80 years old
2	Identified Stanford type A aortic dissection patients
3	Patients who are able to understand the purpose of the study and participate voluntarily and sign informed consent in conjunction with clinical follow-up
Exclusion Criteria	
1	Patients with severe malperfusion syndrome, e.g. coma, paraplegia, progressive liver dysfunction, Gastrointestinal necrosis, extremity necrosis, etc
2	Patients undergoing continuous renal replacement therapy or presenting with renal dysfunction (serum creatinine > 200μmol/L)
3	Patients with severe liver dysfunction (total bilirubin > 205μmol/L)
4	Patients with severe myocardial injury (Troponin I being 100 times larger than upper limit of normal value)
5	Patients with myocardial infarction within 3 months
6	Patients with severe infections that cannot be controlled and induce sepsis, shock or multi-organ failure
7	Patients who are intolerant to anesthesia or cardiopulmonary bypass
8	Patients who underwent surgery within 3 months
9	Patients who underwent thoracic aortic surgery or abdominal aortic surgery previously
10	Patients with active bleeding previously or refusing to receive blood products transfusion
11	Pregnant or lactating patients
12	Patients allergic to contrast agent
13	Patients with a life expectancy of less than 1 years
14	Patients who participated in any on-going clinical trial of drug of medical device before being recruited
15	Patients who are not suitable for this study after evaluation by investigators

Supplemental Table S2: Definition of successful medical device implantation

Successful medical device implantation was identified when meeting all of the following	
1	The delivery system is smoothly deployed into descending aorta
2	The sutureless elastic support annulus and stented graft are located properly and released successfully
3	The delivery system is removed safely and securely; (4) the vascular graft is anastomosed to native vessels completely

Supplemental Table S3: Definition of ischemic hepatitis

Ischemic hepatitis was diagnosed based on all of the following	
1	Rapid and transient increase in either ALT or aspartate aminotransferase AST to a level of more than 10 times the upper limit of normal within 2 weeks postoperatively
2	Mild elevation of the serum bilirubin during rapid-increasing period of ALT or AST, with total serum bilirubin levels typically measuring at less than 3 mg/dL (51.3 μmol/L)

ALT, alanine aminotransferase; AST, aspartate aminotransferase.

Supplemental Table S4: Definition of AKI

AKI was defined as any of the following	
1	Increase in SCr by ≥ 0.3 mg/dl (≥ 26.5 μmol/L) within 48 hours
2	Increase in SCr to ≥ 1.5 times baseline, which is known or presumed to have occurred within the prior 7 days
3	Urine volume < 0.5 ml/kg/h for 6 hours

AKI, acute kidney injury; SCr, serum creatinine.

Supplemental Table S5: Staging of AKI

Stage	SCr	Urine output
1	1.5-1.9 times baseline OR<br>≥ 0.3 mg/dl (≥ 26.5 μmol/L) increase	< 0.5 ml/kg/h for 6-12 hours
2	2.0-2.9 times baseline	< 0.5 ml/kg/h for ≥ 12 hours
3	3.0 times baseline OR<br>Increase in serum creatinine to ≥ 4.0 mg/dl (≥ 353.6 μmol/L) OR<br>initiation of renal replacement therapy OR<br>in patients < 18 years, decrease in eGFR to <35 ml/min per 1.73 m^2	< 0.3 ml/kg/h for ≥ 24 hours OR<br>anuria for ≥ 12 hours

AKI, acute kidney injury; SCr, serum creatinine; eGFR, estimated glomerular filtration rate

Supplemental Table S6: Stages of AD

Time phase	Definition
Hyperacute	Symptom onset within 24 hours
Acute	Symptom onset between 2–7 days
Subacute	Symptom onset between 8–30 days
Chronic	Symptom onset >30 days

Supplemental Table S7: Supplemental preoperative characteristics

Variables	Value (n=10)
Cardiac function	
NYHA class I	0 (0.0)
NYHA class II	0 (0.0)
NYHA class III	0 (0.0)
NYHA class IV	0 (0.0)
Normal	10 (100.0)
Chest pain	8 (80.0)
High blood pressure	
WHO class I	1 (10.0)
WHO class II	1 (10.0)
WHO class III	5 (50.0)
Regular oral medication	6 (60.0)
Smoking	
Packs per day in smokers	1.00 (0.63, 1.00)
Current status	
Never stop	5 (50.0)
Stop within 30 days	0 (0.0)
Stop for more than 30 days	1 (10.0)
TEM classification	
Type A	10 (100.0)
Type B	0 (0.0)
Type non-A non-B	0 (0.0)
E0	0 (0.0)
E1	7 (70.0)
E2	2 (20.0)
E3	1 (10.0)
M0	1 (10.0)
M1-	0 (0.0)
M1+	0 (0.0)
M2-	8 (80.0)
M2+	0 (0.0)
M3-	4 (40.0)
M3+	2 (20.0)

Data are presented as n (%) or median (25% [quartile 1] to 75% [quartile 3]). NYHA, New York Heart Association; WHO, World Health Organization.

Video 1: Detailed operation animation of the Sutureless Integrated Stented graft prosthesis

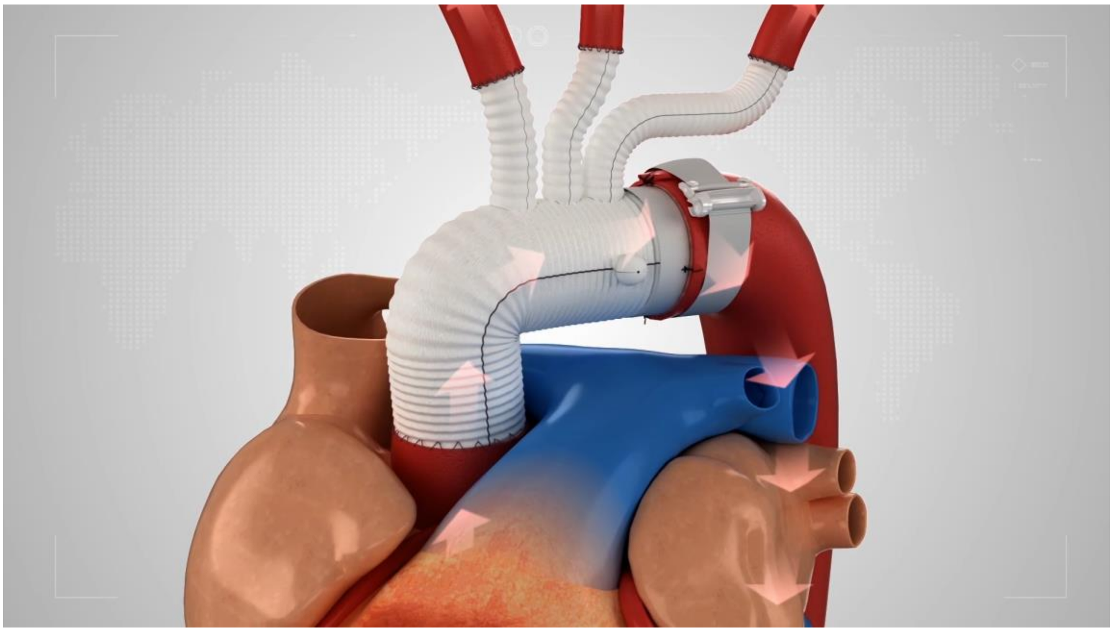

Video 2: Operation video of one acute type A aortic dissection patient and one chronic type A aortic dissection patient implanted with the Sutureless Integrated Stented graft prothesis

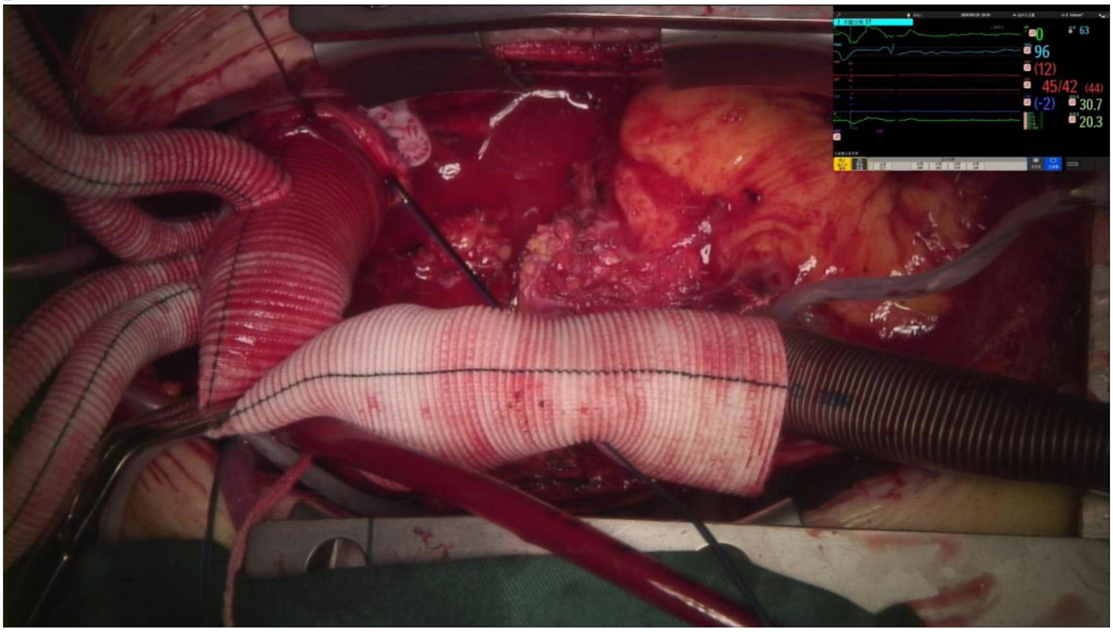

Supplement: Supplementary file 3 [file Data_Sheet_1.PDF]
